# Access, use and perceptions regarding Internet, cell phones and PDAs as a means for health promotion for people living with HIV in Peru

**DOI:** 10.1186/1472-6947-7-24

**Published:** 2007-09-12

**Authors:** Walter H Curioso, Ann E Kurth

**Affiliations:** 1 School of Medicine. Universidad Peruana Cayetano Heredia, Lima, Peru; 2 School of Public Health and Administration, Universidad Peruana Cayetano Heredia, Lima, Peru; 3 Department of Medical Education and Biomedical Informatics, School of Medicine, University of Washington, Seattle, WA, USA; 4 Department of Epidemiology, School of Public Health and Community Medicine, University of Washington, Seattle, WA, USA; 5 Biobehavioral Nursing & Health Systems, School of Nursing, University of Washington, Seattle, WA, USA

## Abstract

**Background:**

Internet tools, cell phones, and other information and communication technologies are being used by HIV-positive people on their own initiative. Little is known about the perceptions of HIV-positive people towards these technologies in Peru. The purpose of this paper is to report on perceptions towards use of information and communication technologies as a means to support antiretroviral medication adherence and HIV transmission risk reduction.

**Methods:**

We conducted a qualitative study (in-depth interviews) among adult people living with HIV in two community-based clinics in Peru.

**Results:**

31 HIV-positive individuals in Lima were interviewed (n = 28 men, 3 women). People living with HIV in Peru are using tools such as cell phones, and the Internet (via E-mail, chat, list-serves) to support their HIV care and to make social and sexual connections. In general, they have positive perceptions about using the Internet,   cell phones and PDAs for HIV health promotion interventions.

**Conclusion:**

Health promotion interventions using information and communication technology tools among people living with HIV in resource-constrained settings may be acceptable and feasible, and can build on existing patterns of use.

## Background

The Peruvian HIV epidemic has largely has been concentrated among men who have sex with men (MSM) and female sex workers (FSW) [1,2]. The seroprevalence rate for MSM is 10–22% [2-4], compared to 0.1–0.4% for the general population [5]. The seroprevalence rate for FSW is 1% [6]. Innovative approaches are needed to enhance adherence to highly active antiretroviral treatment (ART) and to support HIV transmission risk reduction for people with HIV/AIDS. High (ideally > 95%) and sustained adherence to ART is necessary to avoid development of viral resistance. However, average ART adherence among initial cohorts of HIV-positive individuals in some resource constrained settings is only 77% [95% CI 68–85%], as noted in a recent meta-analysis of 27 African studies, and is even lower in resource-rich settings such as the US [7]. In many individuals ART adherence declines over time, and ongoing support is needed. Secondary HIV transmission prevention (i.e., from HIV-positive to HIV-negative individuals) has been under-utilized [8], and evidence-based 'prevention with positives' counseling models [9] have not yet been published in many resource-constrained settings [10,11].

World Wide Web tools [12], personal digital assistants (PDAs) [13], and cell phones [14] have been or are currently being investigated to promote HIV disease self-management. Persons in Peru who are living with, and at risk for, HIV infection also are using these technologies on their own initiative [15]. In Peru, sales of cell phones has jumped from 2.9 million in mid-2003 to 9.8 million by May 2007 [16]. Peru has one of the highest number of Internet users in public places. By January 2006, there were an estimated 10 million Internet users in Peru [15].

Boberg et al [17] suggested that people living with HIV/AIDS have specific needs including contact with other people living with HIV/AIDS, access to social supports to aid decisions around treatment, and access to information about various social services available. Reeves [18] found that the impact of Internet use on coping ability involved three themes: the Internet promotes empowerment, augments social support, and facilitates helping others.

We found no previous published reports on the perceptions among people living with HIV towards information and communication technologies in Peru. The objective of this paper is to report on information and communication technology access, use, and perceptions as a means for health promotion to support treatment adherence and HIV transmission risk reduction among people living with HIV in Peru.

## Methods

We conducted formative research to assess perceptions towards information and communication technologies to support ART adherence and safer sex behaviors. This was done using in-depth interviews with adult HIV-positive people receiving ART and clinical services at two community-based clinics (Impacta, and Via Libre) in Lima, Peru. Both of these clinics primarily serve male clients (100% at Impacta, approximately 80% at Via Libre).

A qualitative interview topic guide was developed based on a published article by Flicker et al. [19], and an instrument previously used among HIV-positive individuals [20]. HIV consultants in Lima reviewed the guide in order to determine its face validity and cultural relevance. Topics covered in the guide pertaining specifically to information and communication technologies are described as follow:

### Computers

How do adult HIV-positive people use computers?

• Do you ever use computers? What for? Frequency?

### Internet

How do adult HIV-positive people use the Internet?

• Do you ever use the Internet? What for?

• Do you know about any sites out there for people living with HIV? Do you use them? For what?

• Where do you access these?

• Do you ever use E-mail? Chat? E-groups? Online support groups? Forums? Blogs? Advantages/Disadvantages? Which ones do you prefer?

### Cell phones

How do adult HIV-positive people use cell phones?

• Do you ever use cell phones? What for? Frequency?

• Do you ever text messaging (Send/receive)?

• Do you ever use the alarm function of your cell phone? Are you currently? For what?

• If we were going to develop an application for people living with HIV in Peru using cell phones – what sort of things do you would like to see? e.g., automatic reminders, prevention messages. Any preference of using your own cell phone? or a new one?

### PDAs

• If we were going to develop an application for people living with HIV in Peru using PDAs (handhelds/minicomputers, what sort of things should we be sure to include? (What content/information would you like to see on the PDA? e.g., info on medications.

• Which type of technology device do you want to use to discuss your HIV health needs? Why? How about confidentiality?

• What sort of things should we include? e.g., counselors, chat with peers.

Inclusion criteria were HIV-positive persons, older than 18 years and receiving ART. Sampling was performed by convenience in two clinics. Participants were demographically similar to those receiving care in both clinics.

Respondents spent approximately one hour for the in-depth interview conducted by an experienced and trained psychologist in each of the clinics (Impacta and Via Libre). After giving consent, all interviews were conducted in Spanish in a private room, and were tape-recorded. Audio files were transcribed and transcripts were reviewed by a Spanish-speaking investigator (WC) for initial text element and key word coding; these codes and categorizations were then reviewed by a second investigator (AK) for final consensus. Data were entered into Atlas.ti qualitative software for theme identification using a content analysis approach [21]. This analysis was intended to understand the information needs, motivations, and behaviors of people living with HIV on ART in Lima [22].

This project and the topic guide used in this study had ethical approval from the University of Washington Human Subjects Division and the institutional review boards of Via Libre and Impacta. All participants signed an informed consent prior to enter to the study. Participants were compensated with 30 soles (about 10 dollars) for travel expenses.

## Results

During March–August 2006, 31 people living with HIV were interviewed, 16 at Via Libre Clinic and 15 at Impacta Clinic. Table 1 provides representative demographic characteristics of HIV positive persons.

**Table 1:** Demographic characteristics of HIV positive persons (n = 31)

	**Mean**	**S.D.**
**Participant age**	36.7	10.7

	**N**	**%**
**Gender**		

**Male**	28	90
**Female**	3	10

**Ethnicity**		
**Mestizo**	25	81
**White**	6	19

**Education**		
**Below high school**	00	00
**High school only**	12	39
**Above high school**	19	61

Findings regarding specific information and communication technologies are reviewed below.

### Internet

Table 2 provides representative verbatim quotes from individuals regarding the perceived advantages and disadvantages of Internet use, for HIV information seeking. For some people, information from the Internet can be overwhelming and may need to be discussed with knowledgeable providers; nonetheless many participants said it was a very important information source for them. A few people 3/31 (10%) reported that they found discussion groups to be useful, as a way to connect with others.

**Table 2:** Representative quotes from people living with HIV regarding the Internet

**Quotes of people having positive experiences**	
**HIV treatment information**	"I look for health information on the Internet such as the evolution of medicines in other countries, side effects, types of medicines and the quality of them; try to keep up-to-date so then I can inform to other people." Male, 38 years
	"I look for information on the Internet to receive some advice, to be prevented of side effects of medicines; how to detect some [HIV-related] diseases, which medicines they take to combat those diseases, types of prophylactic medicines. It's a lot of information." Male, 36 years
	"When I wake up, I turn on my computer, it's the first thing I do. I read some magazines and journals related with HIV... I read all the headlines first. I wish I could read on the Internet 'the cure for HIV was just discovered.' I haven't read something like that yet in the headlines." Male, 36 years
	"I like the forums because you can discuss/propose a topic. For example, I'd like to receive topics of recent updates of the disease/research about HIV. For example, to post something that happened to you and how to deal with." Male, 28 years
	"I think I know quit a bit about the HIV, medicines, side effects, a lot of things. At the same time, I don't wanna miss anything" (smiles).... I even programmed alerts in Google, so I receive information of anything on what's going with HIV. I think it's important the technology because without it right now I could be like a blind fish, not knowing where to go, and following the advice of doctors. I think It's not enough; so for me it's important to be informed." Male, 36 years
**HIV transmission risk reduction information**	"After I was diagnosed with HIV, I looked for information on the Internet on lifestyles, on how to take care on my skin, on how to protect other people." Male, 43 years

**Quotes of people having negative experiences**	

**Overwhelming**	"I get info through the Internet, it's the most important source of information for me. You can find good and important information but also there is a lot of silly information. Internet can inform you but also can confuse you, it could increase your worries.... I discuss what I found on the Internet with my physicians and my psychologist." Male, 36 years
	"When I changed my antiretrovirals, I wanted to know the possible adverse effects of the new medicine (efavirenz). I found on the Internet that it produces nightmares, etc. After reading that, I started having nightmares. So then, I decided NOT to go to the Internet anymore to read about the updates, new adverse events, etc., because you get psycho." Male, 43 years
	"I don't like the Internet because is kind of complicated. For example, in the chat, you can start chatting with one person and then other and other one; and that is what I don't like." Female, 28 years
**Potential for misinformation**	"Anyone can say whatever on the Internet and this is not always true." Male, 35 years
**Waste of time/money**	"I like forums on the Internet because I can read about experiences from others that I might shared with. Unfortunately the forum that I used [name of forum] didn't work very much for me. The first days that I was diagnosed with HIV I posted several questions on the Web, and I never got response. The most I hate is wasting my time, energy and even money, in being connected, and at the end, nobody responds." Male, 36 years

Two participants (6%), both of them MSM, indicated that they use or have used the Internet to see sexual partners. As one 35-year-old man said, "I use the Internet, look, to contact friends. If I wanna meet somebody I go to [name of gay chat rooms]. I contacted people for having sex." Another 43-year-old said, "I heard about people that enter to chatrooms to have sex, a lot. I think 99% of people that do that is to look for sexual contacts. I used to do that, before I was diagnosed with HIV".

In general, participants had positive experiences with the Internet regarding finding HIV treatment information. Some people had negative experiences regarding the Internet being overwhelming, being a potential source of misinformation and thus being a waste of time and money.

### Cell phones

Table 3 provides representative verbatim quotes from individuals regarding the perceived advantages and disadvantages of cell phones. Of 31 people interviewed and currently taking ART, 24 (77%) were using cell phones at the time of the interview. Some of these, 7/31 (23%), already were using the alarms on their cell phones to remind themselves to take their HIV medication. Two participants (6%) reported using their cell phones to send and receive text messages for connecting with others for social and for sexual purposes.

**Table 3:** Representative quotes from people living with HIV regarding cell phones

**Quotes of people having positive experiences**	
**As reminder device for medication & safer sex**	"Using my cell phone is like having another arm. I always use the alarm function of mine [cell phone] to wake up in the mornings and I use it as a reminder [to take my pills]." Male, 39 years old
	"I use the alarm function to take my medicines. I use my cell phone to send and receive SMS. I think cell phones are good because you have an agenda, an alarm, etc." Male, 29 years old
	"I think it's important [to use cell phones to receive prevention messages] because you can be updated and be prevented." Male, 48 years old
	"It has to get to you. Definitively. Even by chance, you always open your SMS messages, even by chance. And you might find something. You have to be obstinate; you have to be very incisive when you leave your clear message. For example: How is going everything? Did you take care of yourself" Did you use your condoms today? It looks like offensive or pushing, but it is a way to attack directly the problem and that people can listen to you. I'd prefer an SMS, and it should be incisive, rather than a pre-recorded voice message." Male, 34 years old
**Confidentiality**	"For me using cell phones is confidential. I don't give my cell phone to anybody." Male, 39 years old

**Quotes of people having negative experiences**	

**As reminder device for medication & safer sex**	"I don't use the alarm function of the cell. I [always] remember to take my medicines. I'd not like to use a cell phone with a recorded voice as reminder. I will not like it. To hear 'you have to take it', no no no; I will feel like a baby." Female, 38 years old
	"I used to use the alarm function of the cell phone at the beginning. Using cell phones as reminders is a good idea. Using cell phones to receive HIV prevention and information messages is a good idea too but up until certain limit because it might be uncomfortable...you know... you are not just living for getting information on HIV... you are doing other things." Male, 35 years
	"It could be useful but mainly for [ART-] naïve patients. For me, it will not work. Now, I automatically know the time of my medication; but it could be certainly useful for naïve patients." Male, 38 years old
**Technological features of cell phone (confidentiality concerns)**	"The problem with cell phones might be with the confidentiality." Male, 34 years old
	"I HATE cell phones. Lots of people offered me, even for free. I think cell phones ruin your privacy. I NEVER had a cell phone. I think I am the only one that doesn't have a cell phone in my group of friends." Male MSM, 36 years

Most of those interviewed, 23/31 (74%), reported their willingness to use cell phones to receive reminder messages for their HIV medication, either by a pre-recorded voice 17/23 (74% of those willing) or by use of short-text messaging, 17/23 (74% of those willing). Not everyone felt the sustained need for using alarm reminders, after learning how to integrate medication-taking into their lives, but saw that this might be useful for ART-naïve patients particularly.

The majority, 25/31 (81%), expressed their interest in receiving messages about their sexual health over the phone, including information about sexually transmitted infections (STIs). Of those, 22/25 (88%) would prefer sexual health messages via text-messaging and 17/25 (68%) via calls with a pre-recorded voice. Furthermore, many said they would like to receive general HIV information via cell phones, including advances in HIV treatment and recent research ("Everything on STDs and HIV/AIDS"). Participants underscored the need for up-to-date information.

In general, people perceived that cell phones were confidential, though some voiced concerns about privacy. Most people said they would prefer to use their own cell phones rather than a new one that might be provided by a research study or a health agency.

### Personal Digital Assistants (PDA)

None of the participants have used a PDA before. The majority, 22/31 (71%), reported that using a PDA might be very good and 8/31 (26%) people reported that using a PDA might be good as a mean for health promotion. The majority, 27/31 (87%) are willing to use a PDA.

Perceptions of advantages were: to provide information (18/31), as a reminder (10/31), to communicate with a doctor (3/31), being confidential (1/31), as a counseling tool (1/31) and to interact with other people (1/31).

Perceptions of disadvantages were: being lost/stolen (5/31), lack of privacy/confidentiality ("somebody might find the PDA in your purse, so it might identify you as HIV positive") (5/31), cost issues (4/31), lack of training or getting familiar with the PDA (3/31), battery issues (2/31), access issues ("you need to come to the clinic to interact with the PDA (2/31) and size issues ("it's big compared with cell phones") (1/31).

## Discussion

Many individuals living with HIV in Peru already use or are open to using the Internet, cell phones, and personal digital assistants that might support intervention delivery for HIV treatment adherence and prevention of secondary HIV transmission (i.e., from HIV positive to HIV-negative partners). Clear advantages were seen to using these devices, among them greater confidentiality as compared to face-to-face interactions (which was mentioned as a plus by 7 out of 30 participants, as opposed to 3/30 who raised concerns about privacy related to use of these tools). In a similar resource-constrained setting, Simoes et al., reported that audio computer-assisted self-interview (ACASI) was perceived as providing the best protection of privacy of participants as compared with interviewer-administered questionnaires [23,24].

Our study population perceived that HIV information is important to their health. Similar results were found in HIV-positive African American and Puerto Rican men who felt that HIV information is vital to their health yet is not readily available in minority communities [25]. In Peru, Internet access is widely available through public centers such as Internet cafes (*cabinas publicas*) – small-scale storefront operations that offer low-cost and reliable connections [15]. We identified one Internet user among the 31 interviewed who didn't want to look for information on the Internet. He found the Internet to be cumbersome with too much, and potentially harmful, information (the patient reported feeling "psycho" when reading the adverse events of the medicine). This characteristic was previously reported by Hogan et al [26]. They surveyed 662 HIV people and found that 31% of people living with HIV agreed that at times it was better not to seek information. In the same survey, Hogan et al. reported that 71% of respondents agreed or strongly agreed that it was easy to feel overwhelmed by HIV information [26].

In general though, our study found that people found useful information on the Internet. Other researchers have reported that people living with HIV who use the Internet for health care indicated that they experienced improved knowledge about the illness, increased skills in coping, and support from others [27]. For example, the anonymity provided by online groups allows discussion of potentially embarrassing topics or otherwise taboo; thus use of online groups increases the possibilities for self-disclosure and encourages honesty and intimacy.

There is great potential to improve health through the use of information and communication technologies in developing countries. The problems of low Internet diffusion and the digital divide are obstacles that resource-constrained countries face in using the Internet for health and development purposes. For those having access the Internet, searching for health information on the Internet can be a difficult experience. Literacy issues might be a problem, as many web pages are written in (often, English-language) text. People may find searching to be time consuming and difficult, and, most frustrating, information may not be in their language. For these reasons, use of scarce resources for information and communication technologies may not necessarily be seen as relevant or appropriate in all settings.

There are some organizations in Peru that offer sexual information via the Internet, suggesting that a user audience is present for information technology-delivered sexual health information. For example, the Peruvian Institute for Responsible Parenthood (INPPARES) has incorporated the Internet into its national sexuality information and counseling service. INPPARES provides information on contraceptive methods, STIs, HIV/AIDS, pregnancy, etc [28]. Academic institutions such as the Universidad Peruana Cayetano Heredia (UPCH) have developed several websites. For example, the RED PREVEN website [29] offers prevention information on HIV/STIs as well as a forum for frequent asked questions. Impulsa SIDA is a project of the Tropical Medicine Institute "Alexander von Humboldt" – UPCH [30] that offers prevention information on HIV/STIs as well as information for people with HIV and their families. Unfortunately, those links did not come up during the interviews. Expansion of Internet and other technologies for partner notification [31], HIV/STI risk reduction, and related sexual health approaches seems opportune. For example, in the recent years, technologies such as cell phones, Internet, email and text messaging have been adopted for partner notification [32]. In the first study addressing the use of the Internet for online partner notification, participants of an Internet chat room were notified via e-mail messages about a syphilis cluster and encouraged to seek medical evaluation. As a result 42% of named partners were notified and tested [33].

There are some limitations of the study. The current study was limited by its use of a convenience sample of people living with HIV/AIDS in an urban population of Lima. The participants in this study cannot be considered representative of people living with HIV/AIDS in Peru. We also assessed Internet use, cell-phone use and PDA use by self-report.

Finally, this is the first study to our knowledge that examined   perceptions regarding Internet, cell phones and PDAs as a means for   health promotion for people living with HIV in Peru. Therefore all of   the study findings require replication.

## Conclusion

This qualitative study suggested that cell phones in particular may be useful and culturally-relevant as a way to support medication adherence and HIV transmission risk reduction among persons living with HIV in Lima. Participants showed enthusiasm for the potential impact of cell phones interventions using voice-recorded reminders and SMS texting as a way to deliver behavioral messages, and most participants expressed interest in participating in such an intervention. Other advantages of cell-phone delivered behavioral support include the fact that in many resource-constrained settings, cell phone communication infrastructure already is in place at a population-level scale, and thus would not need to be built separately to support persons with HIV (or potentially, other chronic diseases). Based on these pilot data, our team is now developing the framework for a cell-phone and a PDA delivered HIV intervention in Lima. The qualitative data we report here can help researchers decide which user-centered information and communication technology devices may be useful to people living with HIV/AIDS in resource-constrained settings.

## Competing interests

The authors declare that they have no competing interests.

## Authors' contributions

WHC and AK designed the study and performed the data analysis, interpretation, and manuscript drafting. Both authors read and approved the final manuscript.

## Pre-publication history

The pre-publication history for this paper can be accessed here:


